# Modeling the expenditure and reconstitution of distance above critical speed during two swimming interval training sessions

**DOI:** 10.3389/fphys.2022.952818

**Published:** 2022-09-26

**Authors:** João Antônio Gesser Raimundo, Rafael Alves De Aguiar, Felipe Domingos Lisbôa, Guilherme Ribeiro, Fabrizio Caputo

**Affiliations:** Human Performance Research Group, College of Health and Sport Science, Santa Catarina State University, Santa Catarina, Brazil

**Keywords:** athletes, performance, critical velocity, critical power, severe domain, aerobic capacity

## Abstract

In swimming, the speed-time relationship provides the critical speed (CS) and the maximum distance that can be performed above CS (D′). During intermittent severe intensity exercise, a complete D′ depletion coincides with task failure, while a sub-CS intensity is required for D′ reconstitution. Therefore, determining the balance D′ remaining at any time during intermittent exercise (D'_BAL_) could improve training prescription. This study aimed to 1) test the D'_BAL_ model for swimming; 2) determine an equation to estimate the time constant of the reconstitution of D' (τD′); and 3) verify if τD′ is constant during two interval training sessions with the same work intensity and duration and recovery intensity, but different recovery duration. Thirteen swimmers determined CS and D′ and performed two high-intensity interval sessions at a constant speed, with repetitions fixed at 50 m. The duration of passive recovery was based on the work/relief ratio of 2:1 (T2:1) and 4:1 (T4:1). There was a high variability between sessions for τD' (coefficient of variation of 306%). When τD′ determined for T2:1 was applied in T4:1 and vice versa, the D'_BAL_ model was inconsistent to predict the time to exhaustion (coefficient of variation of 29 and 28%). No linear or nonlinear relationships were found between τD′ and CS, possibly due to the high within-subject variability of τD'. These findings suggest that τD′ is not constant during two high-intensity interval sessions with the same recovery intensity. Therefore, the current D'_BAL_ model was inconsistent to track D′ responses for swimming sessions tested herein.

## Introduction

Swimming is widely recognized as a popular sport and it has been part of the Olympic program since the first modern Olympic Games in 1896. Most of the swimming events at the Olympic program are performed between 50 and 200 m (or about 21–150 s), demanding a high rate of ATP resynthesis by aerobic and anaerobic energy systems (Capelli et al., 1998; Pyne and Sharp, 2014). During swimming training, sets of interval exercises at low and high intensities are interspersed with relief periods lasting generally less than 60 s (Nugent et al., 2017). However, the relief time is prescribed by coaches with scarce scientific support. Given environmental and technological constraints in swimming, feasible technologies of training prescription and assessment are helpful to athletes, coaches, and sports scientists.

At the beginning of the last century, Hill (1925) observed a hyperbolic relationship between work rate or speed and performance time. This power/speed-time relationship is characterized by two parameters: critical power (CP) or critical speed (CS) demarcating the boundary between heavy and severe exercise domains, and the maximum amount of work/distance that can be performed above CP/CS represented by the mathematical expression W′ or D′, respectively (Poole et al., 2016). Both CP and CS as well as W′ and D′ are analogous but expressed in different units of measurement. Although the precise mechanisms of W'/D′ have remained elusive (Broxterman et al., 2016; Hureau et al., 2016; Poole et al., 2016), the exercise tolerance provides similar amounts of work/distance performed above CP/CS and similar attainment of a critical level of intramuscular phosphocreatine, inorganic phosphate and/or pH (Fukuba et al., 2003; Vanhatalo et al., 2010; Jones and Vanhatalo, 2017). Therefore, to any severe intensity exercise the task failure coincides with the complete depletion of W'/D′ during constant and intermittent exercises, while the replenishing of W'/D′ necessitates a sub-CP/CS intensity (Coats et al., 2003; Chidnok et al., 2012).

Skiba et al. (2012) proposed a mathematical model to determine the balance of W′ remaining at any given time during an intermittent exercise session (W'_BAL_) where some amount of W′ is expended and reconstituted during periods performed above and below CP, respectively. This mathematical model was initially developed for cycling exercise and provides a novel approach for coaches to determine the optimal training intervals and intensity (Skiba et al., 2014a) or for athletes to perform the best pace during a competitive race (Patton et al., 2013). Such a model assumes a linear expenditure and a curvilinear reconstitution of W′ comprising two equations: Eq. 1 determines W'_BAL_ considering the work intensity and duration, the relief intensity and duration, and the time constant of the exponential reconstitution of the W' (τW′), whereas Eq. 2 estimates τW′ to be inserted into Eq. 1. In the second equation, the difference between power output at recovery and CP (D_CP_) is fitted to each relief interval and participant, while the mathematical constants are arbitrary parameters from cycling exercise determined by plotting D_CP_ with actual τW' (found by an iterative process until modeled W'_BAL_ equaled zero at the time to exhaustion) (Skiba et al., 2012). Therefore, in theory, whether CP is unchanged and relief intensity is the same for different interval training sessions, the τW′ should be the same for these exercise sessions regardless of work interval intensity and duration as well as relief interval duration. However, it is unclear whether τW′ remains constant during different interval training sessions with the same work interval intensity and duration as well as relief intensity but different relief interval durations.

$$W^{\prime }bal=W^{\prime }-\overset{t}{\underset{0}{\int }}\left(W^{\prime }\exp\right)\left(e^{-\left(t-u\right)/\tau W^{\prime }}\right)$$ (1)

$$\tau W^{\prime }=546e^{\left(-0.01Dcp\right)}+316$$ (2)

where the W'_BAL_ at any point during a training session or race is the difference between the known W′ and the total W′ expended, W′ equals the subject’s known W′ as calculated from CP model, W’exp is equal to the expended W′, (*t* - *u*) is equal to the time in seconds between segments of the exercise session that resulted in a depletion of W′, τW′ is the time constant of the reconstitution of the W′, and D_CP_ is the difference between the recovery power output and the CP.

Although the W'_BAL_ model has been proposed to characterize the expenditure and reconstitution of W′ during intermittent cycling exercises, there are few studies investigating this model for other exercise modalities (Galbraith et al., 2015; Broxterman et al., 2016). Galbraith et al. (2015) applied the W'_BAL_ model during intermittent running (i.e. D'_BAL_) and showed a D'_BAL_ negative on average (−21.2 m) at interval training session termination. The authors also reported a time constant of the exponential reconstitution of the D' (τD′), determined interactively until modeled D'_BAL_ equaled zero at the time to exhaustion. Apparently the τD′ was lower compared with the previously reported τW′ in intermittent cycling (∼376 vs. 578 s) (Skiba et al., 2012). During severe intensity handgrip exercises, τW′ was affected by different contraction–relaxation cycles, ranging between 580 and 2,450 s (Broxterman et al., 2016). In addition, the authors reported an exponential decay relationship between τW′ and CP (Broxterman et al., 2016). Taken together, these data indicated that τW'/τD′ could be modality-specific and should be directly determined.

Based on the aforementioned statements, the application of the D'_BAL_ model for swimming exercise would be useful as a feasible training prescription tool not requiring any sophisticated apparatus. Therefore, the purposes of this investigation were to 1) test the applicability of the D'_BAL_ model for swimming; 2) determine an equation to estimate τD′ for swimming; and 3) verify if τD′ is constant during two swimming interval training sessions with the same work interval intensity and duration as well as recovery intensity but with different recovery interval durations. We hypothesized that 1) D'_BAL_ could be suitable for swimming exercises; 2) τD′ would be related to CS; and 3) τD′ would be similar between different interval trainings sessions. The implications of confirming these hypotheses for coaches and sports scientists would be a cost-free practical tool to consistently determine optimal intervals and intensities for swimming improving exercise prescription and experimental designs.

## Materials and methods

### Participants

Thirteen male trained swimmers (body mass: 71.8 ± 10 kg, height: 177 ± 7.6 cm, age: 21.3 ± 10 years, arm span: 187.3 ± 10 cm) volunteered for this study. Swimmers took part in regional (*n* = 4) and national (*n* = 9) competitions, had 10.8 ± 5.3 years of experience as competitive swimmers and trained 8.9 ± 2.8 times a week (of which 3.1 ± 1.6 were dry-land exercises) with 23.9 ± 5.4 km of volume per week. Swimmers were specialized in freestyle (*n* = 7), breaststroke (*n* = 4), backstroke (*n* = 1), and butterfly (*n* = 1) at 50–200 m (*n* = 10) and 400–1,500 m (*n* = 3) distance events and completed their best swimming performance last year achieving 510 ± 105 FINA points with classification performance ranked at level 4 (Ruiz-Navarro et al., 2022). Swimmers were free from physical limitations, health problems, or musculoskeletal injuries that could affect the tests, as well as reported not using drugs, medication, or dietary supplements that could have any influence on physical performance. Swimmers or their guardians were informed of the benefits and risks of the investigation prior to signing an informed consent. The study was conducted according to the Declaration of Helsinki and was approved by the Institutional Review Board.

### Study design

**FIGURE 1:**
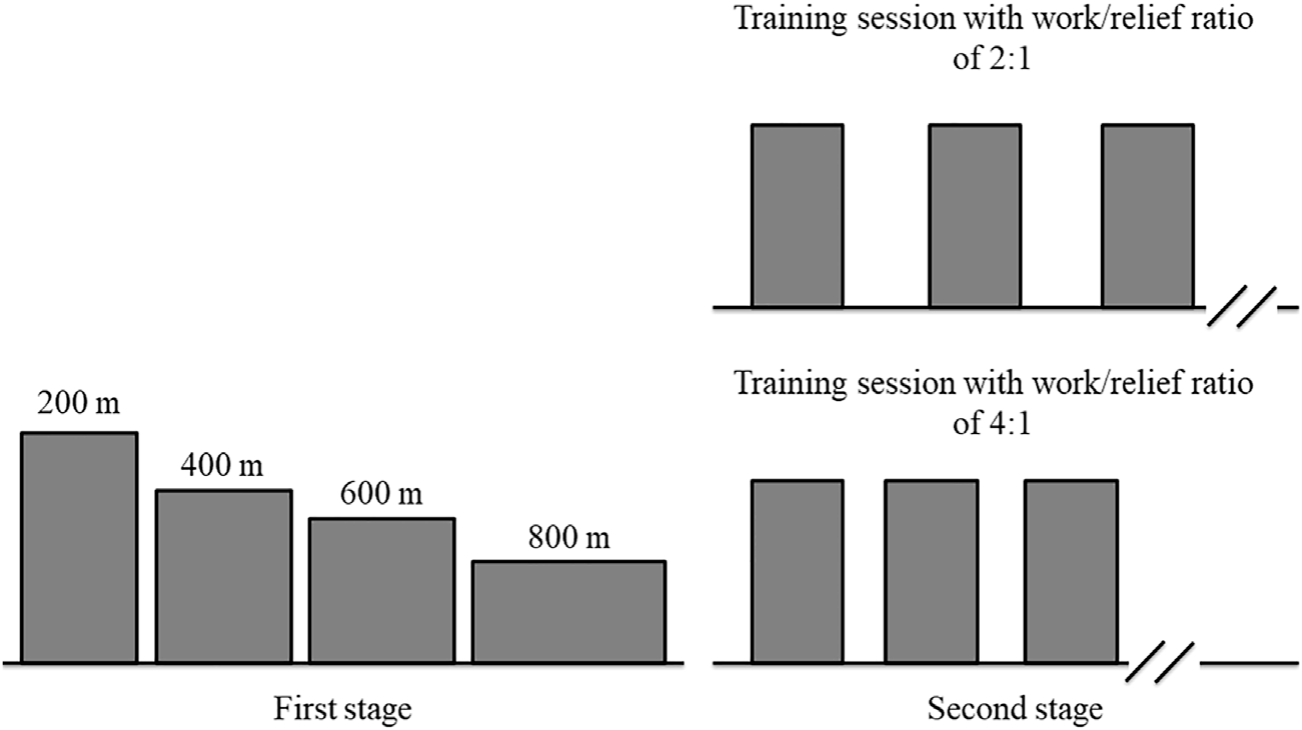
Schematic illustration of experimental design.

Swimmers visited the swimming pool ten times separated by at least 24 h for 3 weeks. All trials were conducted individually in a 25 m indoor pool (28–30°C). Experimental tests were carried out in two stages. The first stage consisted of four randomly performances for CS and D′ determination. The second stage included two high-intensity interval training sessions, in a random order, at a constant speed predicted to lead to exhaustion in 3 minutes during continuous exercise. Between the first and the second stages, the swimmers performed four or five trials to familiarize with this constant speed (Figure 1). All tests were performed in front crawl stroke with a push start and, the swimmers were verbally encouraged to perform the best performance possible (first stage) or continue for as long as possible (second stage). All tests were preceded by a standardized pool warm-up completed in the following order: 300 m freestyle (easy swim); 2 × 100 m freestyle (second faster, higher distance per stroke); 2 × 50 m (25 m kick/25 m easy); 2 × 50 m (25 m drill/25 m easy); 4 × 50 m (25 m at race pace/25 m easy); and 100 m easy swim (Neiva et al., 2014). During the first stage, the race pace warm-up was performed at a speed that swimmers self-selected according to *a priori* expectations about their performances. In the second stage, the race pace warm-up was performed at a constant speed determined for the two interval training sessions. The constant speed was controlled by a pacing device (see below for further details). The warm-up protocol was followed by 10 min of passive rest. All tests started at the same time of day (±1 h) to minimize any effects of diurnal variation (Lisboa et al., 2021). During the study, swimmers were asked to arrive at the swimming pool in a rested and fully hydrated state, abstain from alcohol and strenuous exercise 24 h before testing, and avoid ergogenic aid to enhance performance.

### Critical speed and constant swimming speed

Swimmers were instructed to swim distances of 200, 400, 600, and 800 m as quickly as possible and each performance was recorded at the nearest 0.01 s by a manual stopwatch (Raimundo et al., 2020). These performances were used to calculate the CS (slope) and the D' (*y*-intercept) by applying the distance-time linear regression model (Dekerle et al., 2002). The constant swimming speed that would be predicted to lead to exhaustion in 180 s during continuous exercise was calculated according to Eq. 3:

$$Speed=CS+\left(D^{\prime }/t\right)$$ (3)

where Speed is the target swimming speed, CS is the critical speed, D′ is the distance coursed above CS from distance-time linear regression model, and *t* is the time to exhaustion (set at 180 s in this case).

### High-intensity interval training sessions

Swimmers performed two high-intensity interval training sessions. At each interval training session, the repetitions were fixed at 50 m and were conducted at constant swimming speed. The swimming speed was controlled by matching auditory signals from an electronic speaker (Beat Training & Test, Cefise, Nova Odessa, Brazil) along with nine markers in contrasting colors placed every 2.5 m at the bottom and sides of the 25 m pool. In addition, two investigators walked along the side of the pool at pre-defined pace providing visual feedback when needed. Swimmers were asked to keep their head at the level of the markers for each auditory signal and the test continued until the swimmer’s hand was unable to reach the marker despite strong verbal and visual encouragement (Bentley et al., 2005; Libicz et al., 2005). The exercise repetitions were interspersed by a passive rest with duration based on the work/relief ratio. Thus, the training session was performed with a work/relief ratio of 2:1 (training 2:1; T2:1) or 4:1 (training 4:1; T4:1). For example, a swimmer with a constant swimming speed of 1.44 m s^−1^ completed each repetition in approximately 35 s during both training sessions. Therefore, each relief interval lasted approximately 18 and 9 s in T2:1 and T4:1, respectively. The high-intensity interval training sessions were conducted on different days, performed to exhaustion, and continuously recorded using a camera (Sony DCR-SR68, Tokyo, Japan; 30 Hz) to determine the swimming speed and work and relief intervals durations. This camera was positioned near the edge of the swimming pool perpendicular to the lane. Data from recordings were extracted by a software (Kinovea, v. 0.9.5, MA, United States) and used in all subsequent analyses (i.e. time to perform 50 m and recovery time between repetitions).

### Data analysis

The D′ depletion for each 50 m course and D′ reconstitution during relief intervals were computed to calculate the time course of D′ for the entire interval training session. Data files were analyzed using the continuous equation previously reported by Skiba et al. (2012):

$$D^{\prime }bal=D^{\prime }-\overset{t}{\underset{0}{\int }}\left(D^{\prime }\exp\right)\left(e^{-\left(t-u\right)/\tau D^{\prime }}\right)$$ (4)

where D′ equals the subject’s known D′ as calculated from distance-time linear model, D’exp is equal to the expended D′, (*t* - *u*) is equal to the time in seconds between segments of the exercise session that resulted in a depletion of D′, and τD′ is the time constant of the reconstitution of the D'. Thus, D'_BAL_ at any point during an interval training session or race is the difference between the starting D′ from distance-time linear regression model and the total D′ expended, which is being recharged exponentially when speed falls below CS (Ferguson et al., 2010; Skiba et al., 2012). The τD′ for each participant and interval training session was calculated by an iterative process until modeled D'_BAL_ equaled zero at exhaustion. Actual τD′ found by iterative process from T2:1 and T4:1 were plotted against the CS to determine the better equation to estimate τD'. In the present study, as the work intervals were interspersed with passive recovery periods, CS and difference between recovery speed and CS (D_CS_) were equal.

The τD′ determined for each participant in each interval training session was applied for the other training session (i.e. the individual τD′ determined from T2:1 was applied in T4:1 and vice versa) to predict the time to exhaustion (TTE) and the D'_BAL_ value at the point of interval session termination (D'_END_). As previously noted by Shearman et al. (2016), the D'_BAL_ values can be lower than the D'_BAL_ value at the point of task failure. Therefore, the lowest D'_BAL_ value attained (D'_LOW_) in each training was also determined.

### Statistical analysis

The data are shown as mean ± standard deviation (SD) or 95% confidence interval (CI). Paired *t*-tests assessed possible differences in τD′, D'_END_, D'_LOW_, total D′ expended and reconstituted between training sessions, as well as actual and predicted values. Bland and Altman plots (Bland and Altman, 1986) and coefficient of variation (Hopkins, 2000) examined the consistency of τD′ between training sessions and the predictive ability of the D'_BAL_ model. The within-subject coefficient of variation was calculated by dividing the SD of the differences by the square root of two and dividing the result by the grand mean (τD′) or mean of real value (TTE), and expressed as a percentage (Hopkins, 2000). Statistical significance was accepted at *p* < 0.05. The analyses were performed using Statistical Package for Social Sciences (SPSS) Version 20.0 (SPSS Inc, Champaign, IL). The relationships between τD′ and CS were assessed by linear and nonlinear regressions using GraphPad Prism Version 6.01 (GraphPad Prism; GraphPad Software, San Diego, CA).

## Results

The performances for 200, 400, 600, and 800 m races lasted 136 ± 8, 297 ± 21, 461 ± 35, and 624 ± 45 s, respectively. The distance-time relationship provided average values of 1.23 ± 0.09 m s^−1^ (91.2 ± 2.7% of the 400 m pace) and 33.69 ± 8.65 m for CS and D′, respectively. The goodness of fit of the distance–time relationship was 0.999 ± 0.001 (range: 0.998—0.999). The mean standard error of the estimate were 0.01 ± 0.01 m s^−1^ (1.1 ± 0.7%) for CS and 5.95 ± 3.87 m (18.7 ± 12.4%) for D'. Using Eq. 3, the constant swimming speed that would result in exhaustion in 180 s during continuous exercise was estimated to be 1.42 ± 0.08 m s^−1^ (105.2 ± 2.3% of the 400 m pace). The work interval duration was 35 ± 2 s, while the recovery durations were 18 ± 1 s for T2:1 and 9 ± 1 s for T4:1. The TTE (work plus recovery intervals) were 856 ± 355 s for T2:1 and 301 ± 72 s for T4:1.

**FIGURE 2:**
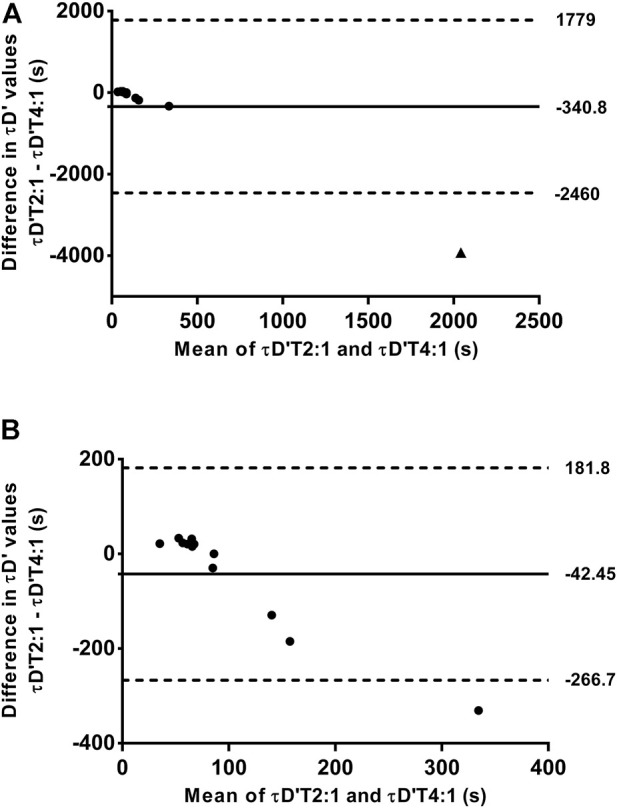
Bland—Altman plots between the time constant of the reconstitution of D' (τD′) found by iterative process from training sessions with work/relief ratio of 2:1 (T2:1) and 4:1 (T4:1). The **(A)** included all swimmers while the **(B)** shows the data analyzed excluding the swimmer 11 (see results session for further details). Horizontal solid line represents the mean difference between τD′ found by iterative process from training sessions with work/relief ratio of 2:1 and 4:1, while horizontal dashed lines represent the 95% limit of agreement. ▲ represents the swimmer 11.

**FIGURE 3:**
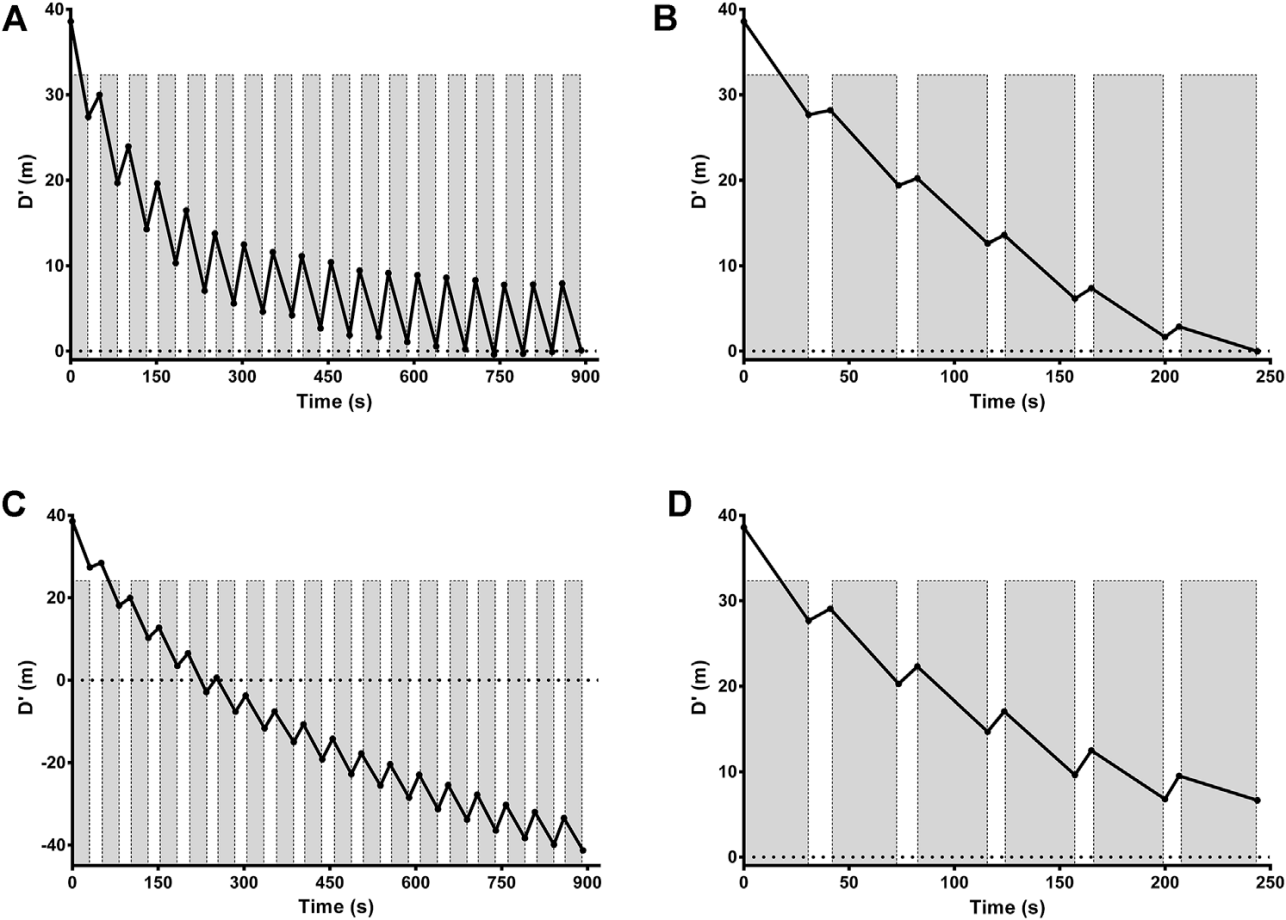
Modeled D'_BAL_ depletion and reconstitution for a representative swimmer in training sessions with a work/relief ratio of 2:1 **(A)** and 4:1 **(B)**. An example for the same representative swimmer of individual D'_BAL_ model when τD′ were inverted in training sessions with a work/relief ratio of 2:1 **(C)** and T4:1 **(D)**. Gray bars indicate work intervals with D′ depletion while white space indicates recovery intervals with D′ reconstitution. Black line shows D′ during depletion and reconstitution cycles. Horizontal dotted line represents D′ equals zero and, in theory, the moment when the swimmer reaches volitional exhaustion.

The mean and individual values of actual τD′ found by an iterative process from T2:1 and T4:1 are shown in Table 1. The τD′ was similar between T2:1 and T4:1 [t (12) = -1.13, *p* > 0.05; 95% CI = -994 to 312 s] but it showed a within-subject coefficient of variation of 306%. Figure 2A shows the bias ±95% limits of agreement of actual τD′ found interactively in T2:1 and T4:1. The D'_LOW_ was lower in T2:1 (−1.86 ± 1.73 m) compared with T4:1 (−0.06 ± 0.23 m) [t (12) = −4.13, *p* < 0.05; 95% CI = −2.74 to—0.85 m]. The D'_LOW_ was lower than zero for twelve swimmers in T2:1 and for two swimmers in T4:1, consequently it was equal to zero for one swimmer in T2:1 and eleven swimmers in T4:1. The Total D′ reconstituted during the passive rests was higher in T2:1 (105 ± 63 m) compared with T4:1 (20 ± 17 m) [t (12) = 5.76, *p* < 0.05; 95% CI = 52–117 m], as well as total D′ expended during exercise was higher in T2:1 (138 ± 64 m) compared with T4:1 (53 ± 18 m) [t (12) = 5.77, *p* < 0.05; 95% CI = 52–117 m]. An example of individual D'_BAL_ model data for a single swimmer in both training sessions is shown in Figures 3A,B (as these τD′ were calculated by iterative processes D'_END_ has to be equal to zero).

**TABLE 1:** The mean and individual values of actual time constant of the reconstitution of the D′ found by an iterative process from T2:1 and T4:1.

Subject	T2:1 (s)	T4:1 (s)	Difference (s)
1	86	86	0
2	75,5	205	129.5
3	73.5	58	15.5
4	81	49.5	31.5
5	68.4	45	23.4
6	70	100	30
7	46	24.5	21.5
8	71.5	51	20.5
9	65	250	185
10	169	500	331
11	78.5	4,000	3,921.5
12	77.7	57	20.7
13	69.5	36.5	33
Mean	79.4	420.2	366.4
SD	28.6	1,083.7	1,072.3

T2:1: Training session with work/relief ratio of 2:1; T4:1: Training session with work/relief ratio of 4:1; SD: standard deviation.

When the τD′ determined for T2:1 was applied in T4:1 and vice versa, the D'_END_ were similar between T2:1 (-10.8 ± 35.8 m; 95% CI = −32.5 to 10.8 m) and T4:1 (−2.6 ± 7.4 m; 95% CI = −7.0 to 1.9 m) [t (12) = −0.71, *p* > 0.05; 95% CI = −33.5 to 16.9 m]. The bias and 95% limits of agreement between actual D'_END_ (i.e. interactively determined and equal to zero) and estimated D'_END_ (i.e. τD′ inverted) was 10.8 m and -59.3–81.0 m for T2:1 and 2.6 m and −11.8–17.0 m for T4:1, respectively.

**FIGURE 4:**
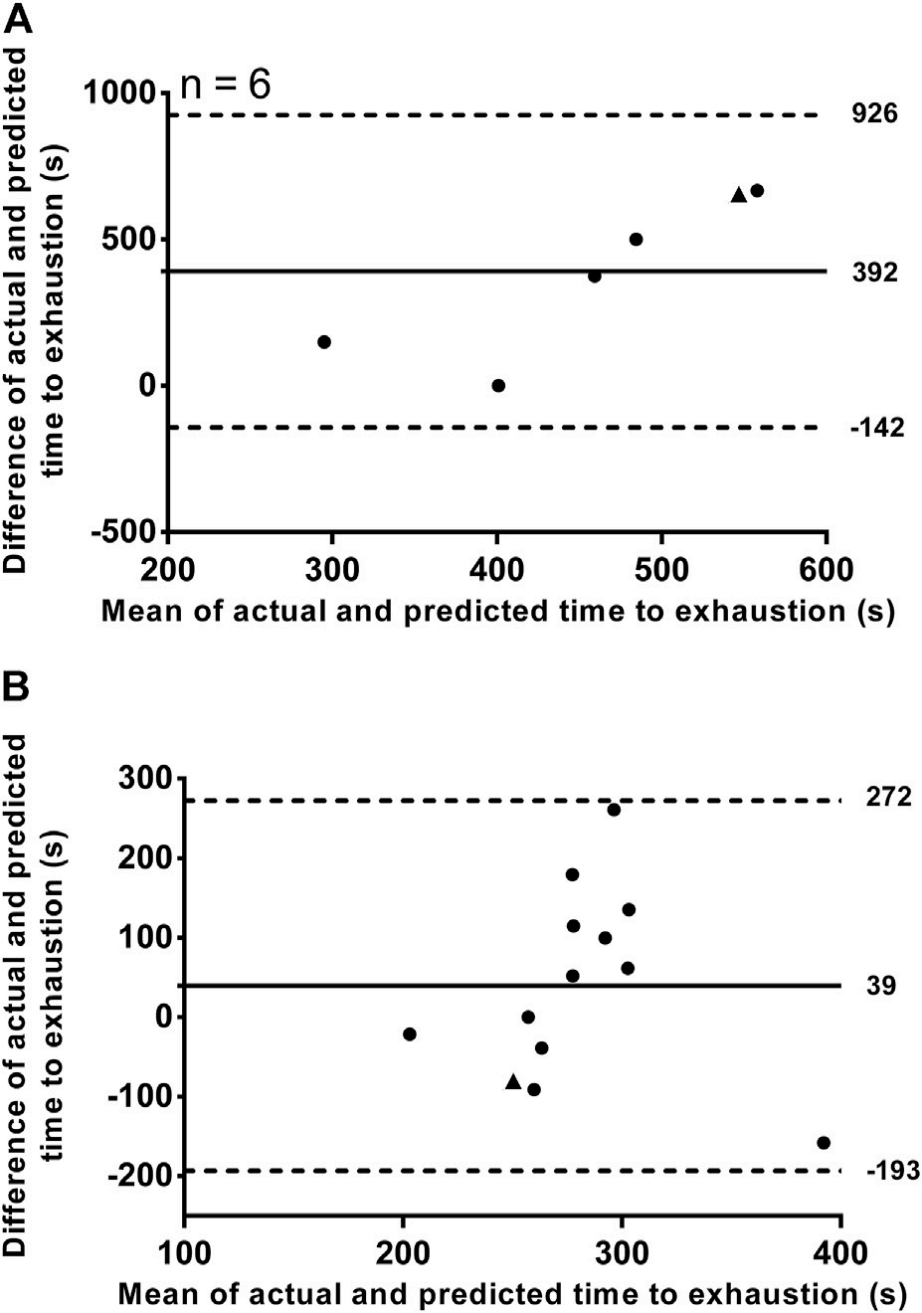
Bland–Altman plots showing individual differences between actual and predicted time to exhaustion plotted against their individual mean values. Training sessions with a work/relief ratio of 2:1 **(A)** and with a work/relief ratio of 4:1 **(B)**. Horizontal solid line represents the mean difference and while horizontal dashed lines represent the 95% limit of agreement. ▲ represents the swimmer 11 (see result section for bias and 95% limit of agreement analyses without this swimmer).

**FIGURE 5:**
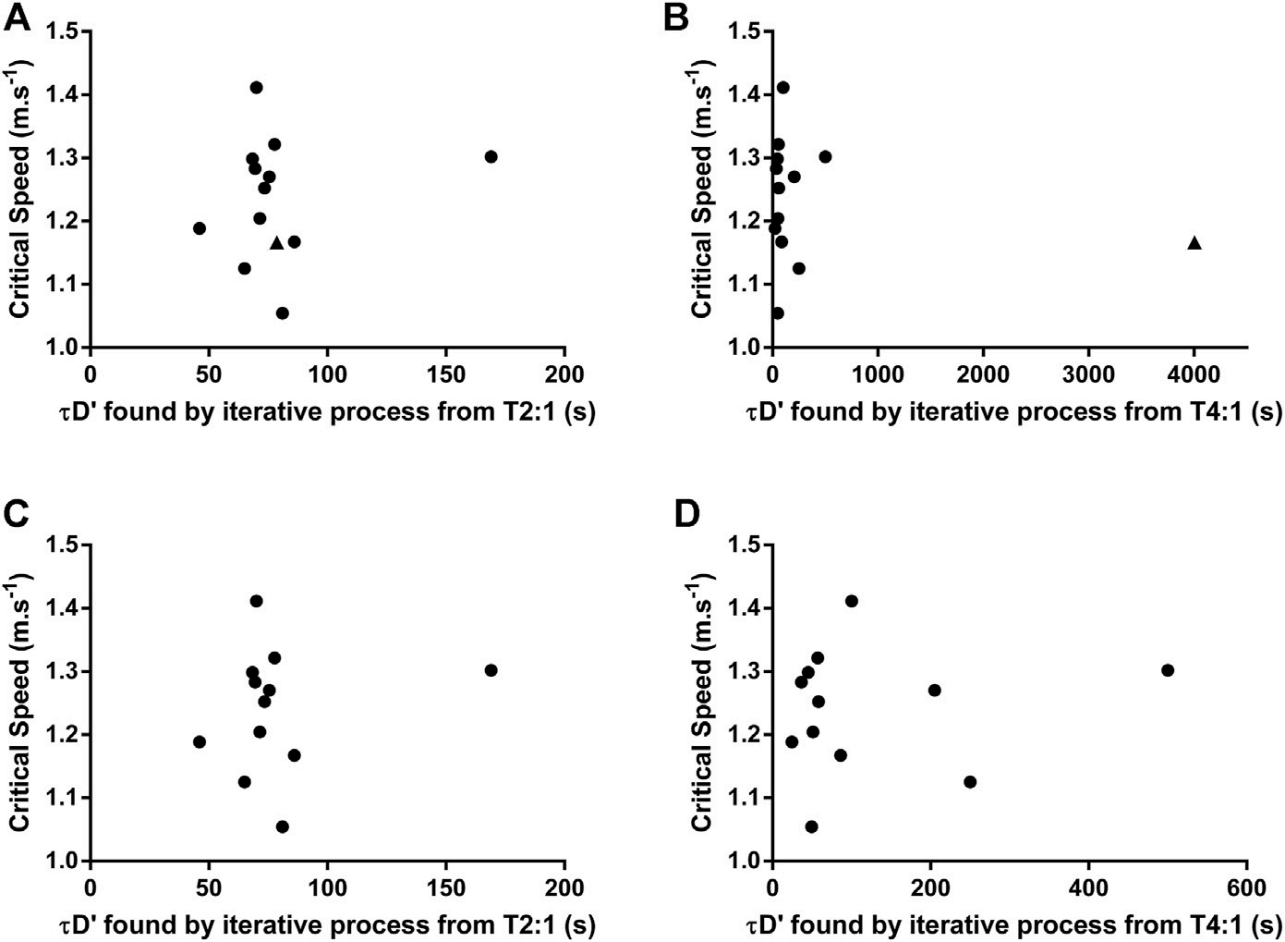
Relationship between Critical Speed and τD′ found by iterative process from training sessions with a work/relief ratio of 2:1 (T2:1) or 4:1 (T4:1). **(A)** and **(B)** show data analysis with all swimmers included. **(C)** and **(D)** show data analysis excluding the swimmer 11 (see results session for further details). ▲ represents the swimmer 11.

An example of individual D'_BAL_ model data when the τD′ was inverted for a single swimmer in both training sessions is shown in Figures 3C,D. It was not possible to predict the TTE with τD′ inverted in T2:1 for seven swimmers because the D'_END_ did not approach zero. In the other six swimmers, the actual TTE was 653 ± 267 s, while the predicted TTE was 261 ± 71 s. The bias and 95% limits of agreement between actual and predicted TTE for T2:1 are shown in Figure 4A and the coefficient of variation was 29%. For T4:1 it was possible to predict the TTE (261 ± 75 s) for all swimmers when applied the τD′ found by an iterative process from T2:1. The bias and limits of agreement between actual and predicted TTE for T4:1 are shown in Figure 4B and the coefficient of variation was 28%. No linear or nonlinear relationships were found between τD′ and CS (all *R* ^2^ < 0.04 or not converged; Figures 5A,B).

The swimmer 11 showed a very different τD′ value in T4:1 (4,000 s) compared with T2:1 and other swimmers (Table 1). This swimmer exhibited no difference during the data collect. Thus, the source for this discrepancy is unclear (e.g. physiological response or random error), but results remain similar when reanalyzed excluding this swimmer. As a result of such reanalyzing, the within-subject coefficient of variation of τD′ was 80.4% with no agreement between the two τD′ values (Figure 2B). The bias and 95% limits of agreement between actual and predicted TTE was 339 s and −186–863 s for T2:1 (*n* = 5) as well as 49 s and −182–281 s for T4:1. Coefficient of variation between actual and predicted TTE was 43% for T2:1 (*n* = 5) and 29% for T4:1. No linear or nonlinear relationships between τD′ and CS were found without the swimmer 11 (all *R* ^2^ < 0.03 or not converged; Figures 5C,D).

## Discussion

This was the first study to model the D′ expenditure and reconstitution during swimming exercise. The main finding of this study was that τD′ is not constant during two similar high-intensity interval trainings, showing high variability between sessions. Thus, when the τD′ determined for T2:1 was applied in T4:1 and vice versa, the D'_BAL_ model was inconsistent to predict the exhaustion of swimmers. In addition, τD′ was not related to CS regardless of the linear or nonlinear equations used. The initial hypothesis has been refuted, suggesting that the current form of D'_BAL_ model is inconsistent to track the dynamic response of D′ during intermittent swimming exercises.

Skiba et al. (2012) were the first to develop the CS/CP model for intermittent exercise using linear expenditure and curvilinear reconstitution of W′ during cycling. According to model theory, the curvilinear reconstitution of the D'/W′ occurs below CS/CP and it is dependent on the difference between recovery intensity and CS/CP (Chidnok et al., 2012; Skiba et al., 2012). Therefore, different training sessions with the same recovery intensity should produce the same τD'/τW'. However, Skiba et al. (2014b) reported that decreasing the recovery duration from 30 to 20 s resulted in an additional reduction of τW′ during cycling exercise with the same recovery intensity. Recently, Caen et al. (2019) and Lievens et al. (2020) confirmed that recovery characteristics can affect W′ reconstitution during cycling exercise. In particular, the model seems to underestimate the reconstitution of W′ after shorter recovery intervals (Caen et al., 2019). In addition, Chorley et al. (2019) and Chorley et al. (2020) reported that the reconstitution of W′ is subject to fatigue following successive bouts of maximal exercise and related to aerobic fitness. Taken together, these results demonstrate that the reconstitution of D'/W′ is more complex than the current model considers (Equations (1) and (4)). As τD′ represents the rate of D′ reconstitution, any physiological changes related to D′ reconstitution should affect τD'. However, the physiological parameters related to D'/W′ have yet to be fully elucidated (Broxterman et al., 2015; Vanhatalo et al., 2016; Raimundo et al., 2019) to improve understanding of D'/W′ reconstitution. In the present study, although not statistically different, τD′ had high variability between training sessions, which resulted in low predictability of D'_END_ and TTE when the τD′ determined for T2:1 was applied in T4:1 and vice versa. Notably, most studies reporting τD′ and the predictive ability of the D'_BAL_ model have only reported systematic changes (Skiba et al., 2014b; Broxterman et al., 2016; Shearman et al., 2016). Despite being an important component for analyzing the robustness of the model, systematic changes do not indicate the consistency of τD′ and D'_BAL_ model as a coefficient of variation and limits of agreement (Hopkins, 2000). Therefore, the dynamics of D′ reconstitution need to be better understood and mathematically described for τD′ to be widely applicable during different swimming interval trainings.

The present study observed that τD′ was not related to CS regardless of linear or nonlinear equations used. On the other hand, τW′ was previously related to D_CP_ or CP in cycling, running, and handgrip exercises (Skiba et al., 2012; Broxterman et al., 2016; Vassallo et al., 2020). As previously mentioned, it is possible that no relationship was found because of the high within-subject variability of τD'. Also, the discrepancies between results might, to a large extent, be due to these studies employing recovery intensity based on different exercise domains (Skiba et al., 2012; Vassallo et al., 2020) or different contraction–relaxation cycles (Broxterman et al., 2016). In accordance, when performing a visual inspection in figures presented by Skiba et al. (2012), Vassallo et al. (2020), and Broxterman et al. (2016), the relationships would likely have a worse or no fit if only one exercise domain were used for recovery intensity (Skiba et al., 2012; Vassallo et al., 2020) or contraction–relaxation cycle (Broxterman et al., 2016). Therefore, although passive rests are usually employed during swimming interval trainings, future studies should relate τD′ and D_CS_ with recovery intensities of different exercise domains.

Considering the aspects mentioned above, the current form of D'_BAL_ model was inconsistent for the swimming interval training sessions tested herein. Before incorporating the D'_BAL_ model into common practices of swimming teams, this model should include other physiological variables as τD′ not being constant during a training session (Chorley et al., 2019). For instance, phosphocreatine seems to be one of the determinants of D'/W' (Miura et al., 1999) as intramuscular phosphocreatine is depleted during high-intensity exercise (Vanhatalo et al., 2010). However, a model for phosphocreatine resynthesis showed a higher concentration of phosphocreatine after exercise, with the phosphocreatine concentration rising ∼5% above that recorded at rest (Nevill et al., 1997). Furthermore, priming exercise can increase CS/CP and/or D'/W' (Miura et al., 2009; Burnley et al., 2011), overestimating the amount of D'/W′ depleted during exercise above CS/CP and underestimating the replenishment during exercise below CS/CP. Collectively, all these physiological factors should be considered to provide a greater practical application of D'_BAL_ model in swimming.

### Limitations

The present study and others reported a high standard error of the estimate for D' (Dekerle and Paterson, 2016), which is usually higher in swimming than in other exercise modes (e.g. running and cycling). While the best practices to determine CS and D′ were used (Muniz-Pumares et al., 2019; Raimundo et al., 2020), we acknowledge that a high standard error of the estimate for D′ can decrease D'_BAL_ model accuracy in swimming. In addition, the swimmers were asked to swim at a constant pre-determined speed, in which there could be some little variation. This possibility was considered prior to the study, we accounted for potential pacing variability by cuing participants according to pre-programmed audio signals, *a priori* familiarization with the protocol, and data analyses performed by video. Lastly, we did not notice any visual difference between pre-determined and real speed during the data collect.

### Practical applications

Based on the findings of the present study, the D'_BAL_ model should be further explored and improved to consistently track the dynamic response of D′ during intermittent swimming exercise. Thus, the D'_BAL_ model needs to take into account other more complex physiological mechanisms that are not currently incorporated as τD′ not being constant during different training sessions with the passive recovery. Hence, athletes and coaches should be aware that the current D'_BAL_ model may not predict the balance of D′ remaining at any given time during swimming interval training. Overcoming these shortcomings, the D'_BAL_ model could contribute to the training prescription in different exercise modalities, especially in swimming which has limitations imposed by the aquatic environment. Lastly, it would be interesting to estimate τD′ based on only passive rests, which are mostly used for swimming.

## Conclusion

In summary, this study confirmed that τD′ is not constant during two swimming interval training sessions, although the same recovery intensity has been used. Consequently, when the τD′ determined for T2:1 was applied in T4:1 and vice versa, the D'_BAL_ model was not able to predict the exhaustion of swimmers. Therefore, the current form of D'_BAL_ model was inconsistent to track the dynamic response of D′ during swimming, at least for the interval workouts tested herein.

## Data Availability

The raw data supporting the conclusions of this article will be made available by the corresponding author on reasonable request, without undue reservation.
